# Schizophrenia: from neurophysiological abnormalities to clinical symptoms

**DOI:** 10.3389/fpsyg.2015.00478

**Published:** 2015-04-20

**Authors:** Anna Castelnovo, Fabio Ferrarelli, Armando D'Agostino

**Affiliations:** ^1^Department of Health Sciences, Università degli Studi di MilanoMilan, Italy; ^2^Department of Psychiatry, University of Wisconsin-MadisonMadison, WI, USA

**Keywords:** psychotic disorders, sleep spindles, parvalbumin-positive interneuron, basket cells, reward system, gamma frequency oscillations, prediction error, predictive coding

## Introduction

Schizophrenia (SCZ) has long been associated with multifaceted dysfunctions and multiple genetic as well as environmental etiological factors (Réthelyi et al., 2013). Therefore, after a century of inconsistent results, the search for a unifying pathogenetic mechanism has become one of the most challenging issues in SCZ research.

During the last decade, a growing literature has pointed to the so-called disconnection hypothesis (Friston, 1998; Tononi and Edelman, 2000; Stephan et al., 2009), i.e., to a defective integration among distributed brain areas, which may lead to a systematic impairment of information processing. EEG is a suitable tool to probe this hypothesis in the time domain, since the EEG oscillatory activity can capture subtle functional changes of underlying neuronal systems with exquisite temporal resolution. Consistent with this prediction, several recent EEG findings have shown abnormalities in SCZ neural oscillations during both wakefulness (Uhlhaas and Singer, 2014) and sleep (Gardner et al., 2014).

Cognitive and theoretical approaches of brain functioning have been used to explain phenomenological features and neural disruptions in SCZ. Since its earliest conceptualizations, abnormalities of self-experience have been identified as a critical feature of the illness (Schneider, 1950). Positive and passivity symptoms in SCZ have been hypothesized to involve a misattribution of self-generated actions, thoughts and percepts to an external agency (Frith, 2005). One possible neurophysiological explanation for this peculiar subjective experience is an aberrant generation of corollary discharge (CD) by efference copy mechanisms (Feinberg, 2011). More recently, predictive coding theories began to add an integrated and structured framework to previous observations (Van de Cruys et al., 2014; Moran et al., 2015).

We herein attempt to reconcile recent major neurophysiological findings with currently established approaches to SCZ psychopathology.

## Predictive coding in schizophrenia

Predictive coding theory considers the brain as learning the statistical regularities in the world and performing inferences using the evidence reported by precision-weighted prediction errors. According to this model, the comparison between bottom-up inputs and top-down predictions yields a prediction error that is weighted in proportion to its expected precision, thus reducing redundancy by removing the predictable components of the input signal. This early theory of sensory processing (Attneave, 1954) was recently implemented by perceptual learning (Friston, 2003) and its application to all brain circuits and cortical microcircuits (Bastos et al., 2012).

Above and beyond the generalization of the model, its use in the context of the reward system seems of particular biological relevance. Indeed, it has been shown that ventral striatum (VS) neurons fire in anticipation of outcomes, and only subsets may also respond during reward consumption (Pennartz et al., 2004, 2011). In particular, dopamine is thought to selectively modulate the strength or gain of associative control over motivated behavior in a regionally specific manner (Pennartz et al., 2011). Crucially, the neuromodulatory effect of dopamine on the gain of postsynaptic responses appears to fit with its putative role in mediating the precision or gain of reward-related prediction errors (Oyama et al., 2010; Schwartenbeck et al., 2014).

A disruption of the delicate balance of precision between beliefs and sensory evidence (Fletcher and Frith, 2009) and the aberrant assignment of salience to elements from one's own experience (Kapur, 2003) have been proposed to underlie the positive symptoms observed in SCZ. Under predictive coding theory SCZ pathology could be described as a failure of neural modulatory gain control that leads to an aberrant weighting of prediction errors and a failure to afford them the precision of salience necessary for perceptual inference and action selection.

In what follows, we will consider the neuromodulatory and synchronous gain mechanisms that may underlie this aberrant processing and subsequent false inference.

## Sleep EEG major findings in schizophrenia

The study of spontaneous neural activity during sleep provides a unique window to investigate the function of the normal and disordered brain. Sleep minimizes possible confounding factors related to waking activities, including changes in the level of attention, decreased motivation or cognitive capacity, and the presence of a variety of symptoms that prevent a reliable task performance (Ferrarelli et al., 2007).

Disruptions in sleep homeostasis, in the sleep/circadian rhythm, as well as in sleep architecture have long been recognized as a symptom in SCZ, and often precede the first clinical breakdown (reviewed in Zanini et al., 2013). More subtle, micro-architectural changes in sleep were recently reported by several different laboratories (reviewed by Gardner et al., 2014).

Whole night deficits in sleep spindles, waxing-and-waning EEG oscillations in the 11–16 Hz frequency range, have been recently reported by several studies. Specifically, spindle density and Integrated Spindle Activity (ISA) were found to be reduced in prefrontal, centro-parietal, and temporal regions (Ferrarelli et al., 2007, 2010). A marked reduction of sleep spindles has been also demonstrated in first-degree relatives and in early course, drug-naïve subjects (Manoach et al., 2014). Conversely, conflicting reports exist regarding slow wave activity (SWA), and no direct data are available to date on sharp-wave/ripple complexes since they cannot be detected with non-invasive scalp EEG recordings.

## The search for the missing link: PV interneurons

Sleep spindles are generated in the thalamic reticular nucleus (TRN), a thin sheet of cells surrounding the anterolateral part of the thalamus (Halassa et al., 2011). The TRN is entirely composed of a rather heterogeneous population of parvalbumin immunoreactive GABAergic neurons (PV+) (Celio, 1990). Several line of evidence currently converge on the implication of PV+ neurons in SCZ. Post-mortem studies revealed GABAergic alterations, in particular in PV+ and calretin neurons (e.g., Beasley et al., 2002) in schizophrenia patients. Moreover, abnormal amplitude and synchrony of oscillatory activity, mainly frontal and at high (gamma) frequencies, have been found in SCZ, during task-related, spontaneous neuronal activity (Uhlhaas and Singer, 2013), as well as after transcranial magnetic stimulation (Ferrarelli et al., 2012; Rogasch et al., 2014). Gamma frequency (30–80 Hz) oscillations require the synchronized inhibition of neighboring populations of pyramidal neurons by the subclass of cortical PV+GABA interneurons (Sohal et al., 2009).

Finally, some animal models have given preliminary support to the hypothesis of a deregulation of PV+ neurons in SCZ (e.g., Carlson et al., 2011; Phillips et al., 2012; Kaalund et al., 2013). PV+ neurons can be found throughout the CNS (Celio, 1990), including the TRN, the hippocampus and the neocortex. They selectively express high levels of the PV Ca^2+^-binding protein acting as a “slow Ca^2+^ buffer” to modulate Ca^2+^ cytosolic homeostasis, short action potential duration and a fast-spiking action potential phenotype. The buffering capacity of PV may protect from Ca^2+^-mediated excitotoxic insult (Figueredo-Cardenas et al., 1998).

Optogenetic and pharmacogenetic approaches have begun to provide insight into the function of PV+ interneurons not only at the cellular, but at both the network and behavioural levels (Hu et al., 2014). PV+ interneurons play a major role in network oscillations (Bartos et al., 2007), and are implicated in perceptual discrimination (e.g., Lee et al., 2012), attention (Zikopoulos and Barbas, 2006), in the regulation of plasticity and learning (e.g., Donato et al., 2013), as well as in reward-related behavior (Sparta et al., 2014).

Finally, PV+ neurons involvement in SCZ is in accordance with the generic role of cortical gain and excitation/inhibition balance, that has been recently proposed to underlie false inference in SCZ (e.g., Adams et al., 2013; Jardri and Denève, 2013). PV+ neuron dysfunction can be primary or secondary to the dysfunction of other pathways and neuromodulators (for a review, Lewis et al., 2012), genetically inherited or environmentally induced (Jiang et al., 2013; Stansfield et al., 2015). While PV dysfunction has recently been proposed as a substrate for cognitive dysfunction in SCZ (e.g., Lewis, 2014), we here focus on its possible connection to the development of positive symptoms and to current theoretical approaches that attempts to explain the complex phenomenology of the disorder.

## Reconciling theory and physiology

The hypothesis of functional impairment of PV+ neurons doesn't only justify the established deficit in sleep spindles, but also leads to speculation over abnormalities in other functional circuits and their possible correlations with neurophysiological findings and symptoms in SCZ.

The TRN, situated in a strategic position between the neocortex and the thalamus (Pinault, 2004), is related to sensory gating in the thalamocortical and corticothalamic axes (Jones, 2002), as well as in the modulation of attention (Zikopoulos and Barbas, 2006). PV deficiency affects the dynamics of burst discharges of TRN cells, which in turn regulate the activity in the thalamocortical circuit (Albéri et al., 2013). TRN tonic activity during wake inhibits spontaneous background activity in specific thalamocortical reley nuclei, probably under the control of attentional mechanism from frontal and limbic structures, and are implicate in lateral inhibition, optimizing responses to sensory stimulation and their transfer to the cortex (Pinault and Deschênes, 1998; Hartings et al., 2003). A deficit of the TRN leads to loss of sensory-specific inhibition, which results in an increase of spontaneous background activity and a decrease of lateral inhibition in specific thalamic nuclei, thus resulting in a reduction in the signal-to-noise ratio or precision of thalamic relays (Ferrarelli and Tononi, 2011).

Abnormal sensory experience, which characterizes the prodromal phase of SCZ and gives rise to hallucinations, would occur when sensory inputs fail to adequately modulate thalamocortical activity (Behrendt, 2006). It has already been suggested that a deficit in TRN can lead to impaired CD mechanisms through a disruption of the integrative function of corticothalamocortical circuits mediated by the TRN (Vukadinovic, 2011). A deficit in the activity of the TRN would therefore produce abnormal sensory feedbacks along with impaired efferent copies from the motor and associative to the sensory cortices, which would generate an imbalance between predicted and actual feedbacks as well as a reduced sense of agency (Vukadinovic, 2011).

The role of DA in generating positive symptoms of SCZ has been hypothesized since the serendipitous discovery that D2 blocker compounds have strong antipsychotic efficacy in SCZ patients (Howes and Kapur, 2009). Furthermore, Dopamine (DA) receptors (D4) have been established on GABAergic interneurons in the cerebral cortex, the HC and the TRN (Mrzljak et al., 1996), but the neural circuits and the effects of dopamine on PV positive TRN neurons are still not well characterized. Additionally, the reduction of vHC PV expression has been found to increase DA activity in the ventral tegmental area (VTA) and behavioral hyper-locomotor-responsivity to amphetamine in awake rats (Boley et al., 2014) via a multi-synaptic pathway (Lodge and Grace, 2007, 2011). In the case of SCZ a PV+ neuron dysfunction may lead to an increased number of DA neurons in VTA spontaneously active, thus affecting the ability of the cortex to appropriately regulate the gain of incoming stimuli. The same salience is therefore assigned to all stimuli, leading to a disruption of the prediction error weighting in systems responsible for action selection and perceptual synthesis. This provides a simple explanation for false inference implicit in symptoms like hallucinations and delusions (Adams et al., 2013; Fogelson et al., 2014).

Finally, PV+ neuron deficits also suggest the presence of other subtle abnormalities in neural oscillations in SCZ, like an alteration in hippocampal ripples. Basket PV+ cells in the HC fire at high frequency and are phase-locked to ripple oscillations (140–200 Hz), providing an inhibitory temporal structure for large populations of pyramidal cells, and possibly contributing to the synchronization of the entire network (Klausberger et al., 2003, 2005; Fuchs et al., 2007; Klausberger and Somogyi, 2008; Rácz et al., 2009). Importantly, hippocampal ripples occur during SWS and consummatory behaviors (Buzsáki et al., 2003), the same pattern of activation observed in the VS.

This is consistent with the Reward Activation Model (RAM, Perogamvros and Schwartz, 2012), in which the reward system plays a central role not only during wakefulness, but also during sleep and dreaming. In particular, HC and VS have been hypothesized to act in conjunction to link memory traces to a motivational value, possibly through hippocampal ripples during NREM sleep.

## Conclusions

We briefly described an approach to SCZ encompassing theoretical models, including disconnection hypotheses and predictive coding abnormalities, as well as neurophysiological findings, from sleep abnormalities to psychopathological signs observed during wakefulness. We suggest that sleep spindles and waking gamma deficit support a dysfunctional role of thalamic and cortical PV+ neurons as a common pathway, either primary or secondary, in SCZ disease, which may explain both cognitive deficits and positive symptoms observed in these patients. Specifically, dysfunction in hippocampal and thalamic PV+ neurons together with more subtle alteration in DA-regulated hippocampal-limbic circuits warrant further investigation and may provide further support to the idea of a disruption in **salience or precision** in SCZ during both wake and sleep. In sum, we believe that the integration of theoretical models and physiological findings will enrich both fields and may lead to the discovery of novel therapeutic targets for patients with SCZ.

### Conflict of interest statement

The authors declare that the research was conducted in the absence of any commercial or financial relationships that could be construed as a potential conflict of interest.
